# Epithelial ovarian carcinoma metastasizing to the pancreas: An uncommon diagnosis with EUS-guided fine-needle biopsy

**DOI:** 10.1097/eus.0000000000000202

**Published:** 2026-07-15

**Authors:** Stefano Rizza, Paola Calamaro, Valentina Tuninetti, Francesco Maria Stalla, Alessandro Lavagna, Marco Daperno

**Affiliations:** 1 Gastroenterology and Digestive Endoscopy Unit, Ordine Mauriziano Hospital, Turin, Italy; 2 Pathology Unit, Ordine Mauriziano Hospital, Turin, Italy; 3 Department of Oncology, University of Turin, Medical Oncology, Ordine Mauriziano Hospital, Turin, Italy.

Pancreatic metastases are rare and represent 2% of all pancreatic neoplasms.^[1]^ Epithelial ovarian carcinoma metastasizing to the pancreas is reported in very few cases, with an overall incidence of 3.8%.^[2–5]^ We report a case of a solitary pancreatic metastasis from a high-grade serous papillary ovarian carcinoma, occurring 9 years after the diagnosis of the primary cancer.

A 56-year-old woman was diagnosed in April 2016 with stage IV ovarian cancer. Initial computed tomography showed bilateral ovarian lesions inseparable from the uterus and sigma, omental nodulations, and signs of peritoneal carcinomatosis. The patient underwent 4 cycles of neoadjuvant chemotherapy with carboplatin plus paclitaxel and subsequent debulking surgery (hystero-annexectomy, splenectomy, left diaphragmatic peritonectomy, removal of glissonian nodes, hepatic-hilar lymphadenectomy, rectal resection plus lymphadenectomy) followed by 2 further platinum-based chemotherapeutic cycles. In November 2018, for hepatic progression (lesions in segments 3–4), a new cytoreductive surgery was performed with liver resection and hyperthermic intraperitoneal chemotherapy. From 2020 until January 2024, she underwent several chemotherapy regimens with platinum-based chemotherapy followed by niraparib maintenance, doxorubicin plus trabectedin, and carboplatin plus gemcitabine. In February 2025, computed tomography scan showed a 30-mm hypodense lesion of the pancreatic body, uncertain whether primary or secondary [Figure 1]. EUS confirmed a hypoechoic, inhomogeneous, poorly vascularized, nodular lesion, with high stiffness on elastosonography: fine-needle biopsy was performed using a 22-G needle (SharkCore, Beacon Endoscopic/Medtronic, Newton, MA, USA), obtaining adequate material in 2 passes [Figure 2]. The histopathological and immunohistochemical analysis described positivity for WT1 and CA-125 with negativity for CK-20 and CDX-2 [Figure 3]. Based on these findings, a final diagnosis of pancreatic metastasis from high-grade serous papillary ovarian carcinoma was made.

**Figure 1.**
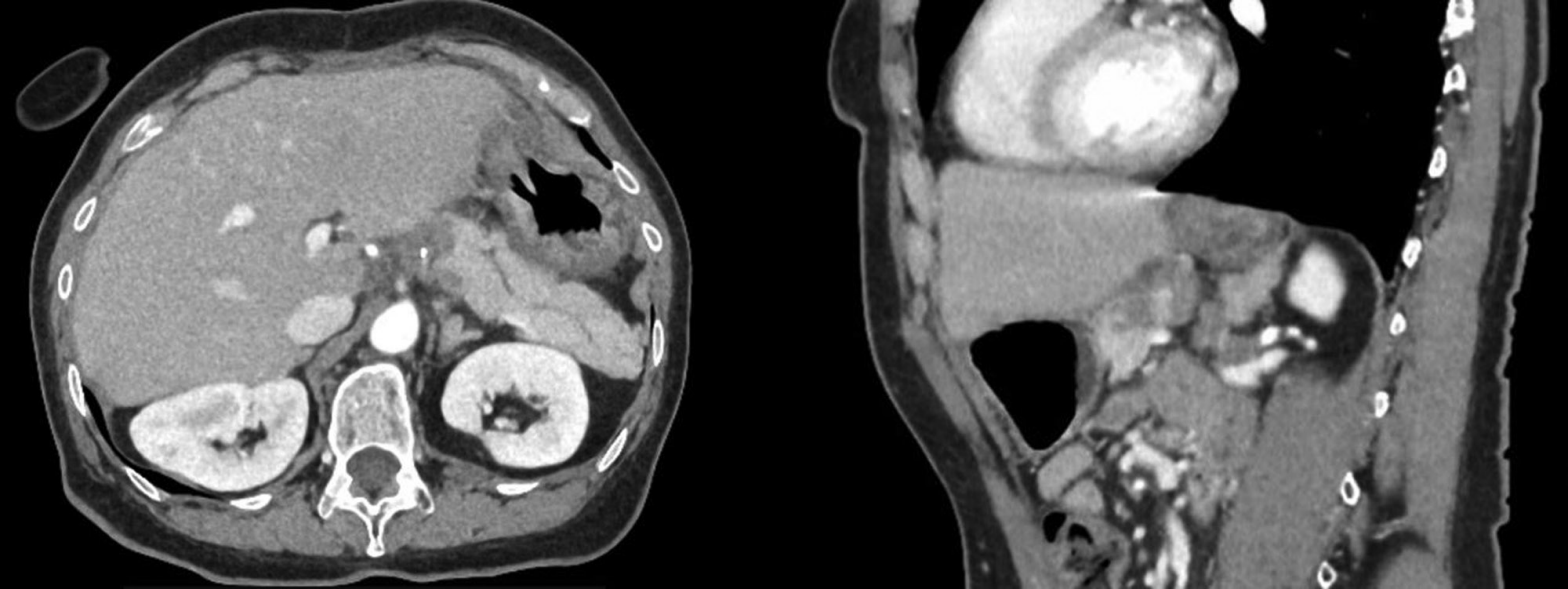
Abdominal CT showing a hypodense lesion of the pancreatic body. CT, computed tomography.

**Figure 2.**
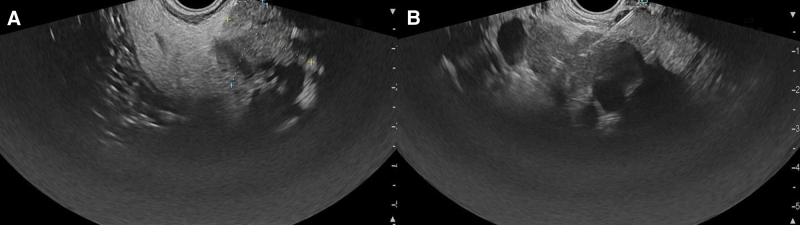
EUS imaging (A) and FNB of the lesion (B). FNB, fine-needle biopsy.

**Figure 3.**
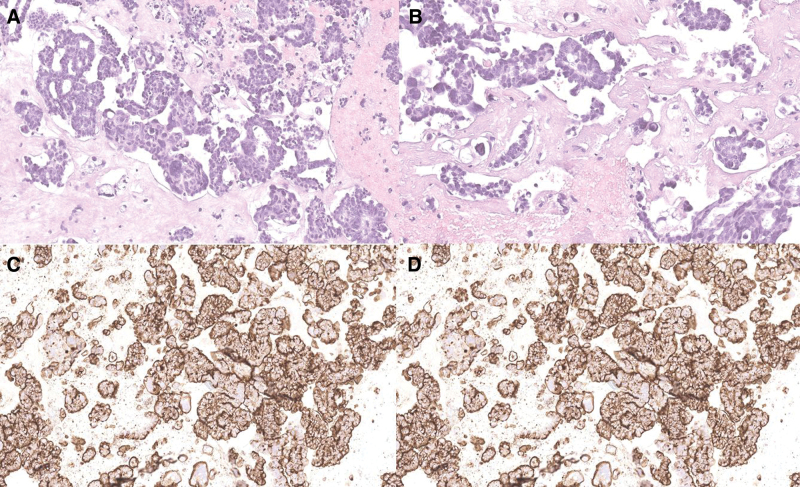
Histological section (hematoxylin and eosin stain) of the pancreatic metastasis (×20 and ×40, A and B) and immunohistochemistry showing positivity for WT1 (×20, C) and CA-125 (×20, D).

In a previous history of nonpancreatic neoplasms, ovarian cancer metastases should also be kept in mind, although very rare. Ovarian cancer can metastasize through the lymphatic, hematic, and intraperitoneal routes but also by direct extension from the retroperitoneum, as probably occurred in this case.

Fine-needle biopsy can provide high-quality tissue cores with preserved architecture and, in conjunction with cytological and immunohistochemical staining, has invaluable diagnostic power in identifying such uncommon lesions.

## Ethical Statements

The authors declare that all procedures performed in this study were conducted in accordance with the ethical standards of the institutional and/or national research committee and with the principles of the Declaration of Helsinki and its later amendments. Informed consent was obtained from the patient for the publication of her information and imaging.

## Conflicts of Interest

The authors declare no conflicts of interest.

## Author Contributions

Conceptualization: S. Rizza. Writing-original draft preparation: S. Rizza and V. Tuninetti. Preparing figures: S. Rizza and P. Calamaro. Data Collection: S. Rizza, V. Tuninetti, and F. Stalla. Writing-reviewing and editing: S. Rizza, V. Tuninetti, A. Lavagna, and M. Daperno. All authors reviewed the manuscript.

## Data Availability Statement

No datasets were generated or analyzed during the current study.
